# Coordinated Optimal Control of AFS and DYC for Four-Wheel Independent Drive Electric Vehicles Based on MAS Model

**DOI:** 10.3390/s23073505

**Published:** 2023-03-27

**Authors:** Niaona Zhang, Jieshu Wang, Zonghao Li, Nan Xu, Haitao Ding, Zhe Zhang, Konghui Guo, Haochen Xu

**Affiliations:** 1 School of Electrical and Electronic Engineering, Changchun University of Technology, Changchun 130000, China; 2 State Key Laboratory of Automobile Simulation and Control, Jilin University, Changchun 130000, China

**Keywords:** four-wheel independent drive electric vehicle (4WIDEV), multi-agent systems (MAS), dynamic sliding mode, distributed model predictive control (DMPC), active front steering (AFS), direct yaw moment control (DYC)

## Abstract

The problem that it is difficult to balance vehicle stability and economy at the same time under the starting steering condition of a four-wheel independent drive electric vehicle (4WIDEV) is addressed. In this paper, we propose a coordinated optimal control method of AFS and DYC for a four-wheel independent drive electric vehicle based on the MAS model. Firstly, the angular velocity of the transverse pendulum at the center of mass and the lateral deflection angle of the center of mass are decoupled by vector transformation, and the two-degree-of-freedom eight-input model of the vehicle is transformed into four two-degree-of-freedom two-input models, and the reduced-dimensional system is regarded as four agents. Based on the hardware connection structure and communication topology of the four-wheel independent drive electric vehicle, the reduced-dimensional model of 4WIDEV AFS and DYC coordinated optimal control is established based on graph theory. Secondly, the deviation of the vehicle transverse swing angular velocity and mass lateral deflection angle from their ideal values is oriented by combining sliding mode variable structure control (SMC) with distributed model predictive control (DMPC). A discrete dynamic sliding mode surface function is proposed for the ith agent to improve the robustness of the system in response to parameter variations and disturbances. Considering the stability and economy of the ith agent, an active front wheel steering and drive torque optimization control method based on SMC and DMPC is proposed for engineering applications. Finally, a hardware-in-the-loop (HIL) test bench is built for experimental verification, and the results show that the steering angle is in the range of 0–5°, and the proposed method effectively weighs the system dynamic performance, computational efficiency, and the economy of the whole vehicle. Compared with the conventional centralized control method, the torque-solving speed is improved by 32.33 times, and the electrical consumption of the wheel motor is reduced by 16.6%.

## 1. Introduction

Four-wheel independent drive electric vehicles (4WIDEV) have the advantages of a compact powertrain, high transfer efficiency, space-saving all-wheel drive, and fast torque response [1]. Since the four-wheel motors are mounted on each of the four wheels, this facilitates independent decoupling control of each wheel’s torque [2]. 4MIDEV is a typical overdrive system [3,4] with more actuators than control system degrees of freedom [5,6,7]. Under steering conditions, different control distribution methods yield different torque distribution results when four-wheel torque multi-objective optimization is performed to improve the stability and economy of 4WIDEV [8].

Currently, many researchers have proposed several control theories and methods, such as fuzzy control, sliding mode variable structure control (SMC), model predictive control (MPC) [9] reinforcement learning [10], and other algorithms. Ying adopts the fuzzy control method for the controller of 4MIDEV to generate optimal regenerative braking torque to improve safety and economy during vehicle deceleration [11]. Lin addressed the stability of 4WIDEV with energy loss problems and proposed an integrated framework that considered tire sliding energy loss and lateral stability control. The upper controller uses a PID speed-tracking controller and a terminal sliding mode controller, and the objective function of the lower controller is the minimum tire slip energy consumption [12]. Although fuzzy control has strong robustness and does not need an accurate mathematical model, its simple fuzzy processing of information will lead to the reduction of system control accuracy and poor dynamic quality, and the stability of electric vehicles is an important factor to measure the dynamic performance of vehicles, so fuzzy control is not suitable for this study. Yue used a model-free adaptive sliding mode control in the upper controller to estimate the required yaw moment, and in the lower controller, Yue used the seeker optimization algorithm (SOA) for torque distribution, which ensured the stability and energy-saving characteristics of the vehicle [9]. Sliding mode control can overcome the uncertainty of the system, and has strong robustness to disturbance and modeling dynamics, especially for the control of nonlinear systems. However, when the state trajectory reaches the sliding mode surface, it is easy to generate buffeting, and buffeting is difficult to eliminate. Wang established a 2-DoF vehicle model and path following the error model to obtain the desired yaw rate through inversion, used MPC to track the desired yaw rate and additional yaw moment, and obtained the optimal front wheel steering angle and additional yaw torque to ensure the path following and vehicle stability of the whole vehicle [13]. Jing used MPC to coordinate the AFS and DYC systems to ensure vehicle stability and minimize energy consumption to reduce the large additional yaw moment of the vehicle under high-speed cornering conditions [14]. Li proposed an effective two-level optimal torque control distribution method to adjust the weight coefficients of the objective function in real time in the second-level distribution control strategy, thus ensuring vehicle handling stability under various attachment conditions [15]. Zhai adopted an adaptive two-layer energy-saving torque distribution algorithm in the lower controller and used the friction circle constraint as the constraint for judging whether to switch the algorithm to ensure a more stable and energy-saving steering operation of the vehicle [16]. References [13,14,15,16] all adopt the MPC control method. MPC has the advantages of good control effect and strong robustness, which can effectively overcome the uncertainty, nonlinearity, and parallelism of the process, and can easily handle various constraints in the controlled variables and the control variables. However, the MPC solution process is mainly aimed at the large matrix inverse calculation, but because the four-wheel independent drive electric vehicle has the characteristics of complex and nonlinear modeling, it will lead to a slow solution speed of MPC, and it is easy to fall into the local optimal solution rather than the global optimal solution. To solve this problem, more and more researchers are now turning their attention to distributed model predictive control. Pi proposed a 4MIDEV energy management method based on DMPC, which took driving/braking deviation, trajectory deviation, and energy consumption as performance indicators for torque distribution [17]. Tang proposed a distributed control architecture that treats each wheel model as a multi-agent and uses the DMPC approach to improve the flexibility and robustness of the system, providing a new perspective on controller design for conventional vehicles [18]. Yin’s team proposes a distributed and coordinated control architecture for 4WIDEV AFS and DYC based on the MPC control method, which considers AFS and DYC as multi-agents to improve the lateral stability of the vehicle [19]. Zhang proposed a multi-objective optimal torque coordination control method for ABS and AFS based on multi-agent DMPC. The four wheels and the center of mass of the vehicle are regarded as multi-agents, and the DMPC method is used to realize that the vehicle follows the ideal values of slip rate, yaw rate, and center of mass slip angle, to improve the braking safety and handling stability of the vehicle [20]. Among them, the DMPC controller has been widely used in intelligent and electric vehicle tracking for its advantages of online optimization, flexible structure, clear constraint solution, and high control efficiency, and has improved the vehicle’s active safety and economy.

The existing control framework is roughly divided into three types: hierarchical centralized control framework [9,11,12,15,16,21,22], distributed structure control framework [17,18,19], and integrated distributed structure control framework [20]. In these three frameworks, the distributed structure framework has the advantage of reducing model complexity and improving control efficiency. So far, most of the existing 4WIDEV lateral stability control methods are layered centralized control architectures, and integrated control architectures have not been considered much in 4WIDEV lateral stability control methods. Since the centralized controller is highly dependent on the vehicle platform, any changes to the actuators and the complexity of the model will lead to system redesign. Therefore, applying the integrated distributed control architecture to 4WIDEV can achieve model dimensionality reduction of complex systems, reduce computational effort, and improve control efficiency with higher flexibility and fault tolerance [23,24]. Therefore, this paper adopts an integrated distributed control framework. Because SMC has the advantages of fast response, strong anti-interference ability, and little dependence on system parameters, more and more scholars have begun to study the lateral stability control strategy of 4WIDEV based on DMPC and SMC in recent years. Benefiting from the DMPC rolling optimization mechanism and the improvement of the SMC robustness, the controller can consider the state trajectory in the future time domain in advance and optimize it, improving the control optimality and ensuring the robustness of the system. Chen and Wang [25] proposed a hierarchical control structure based on SMC and an adaptive energy conservation control assignment (A-EECA) scheme for tracking the driving trajectory of a 4WIDEV and achieving optimal energy consumption. At present, how to enhance the robustness of the whole vehicle system while ensuring vehicle handling stability has not yet attracted sufficient attention from the underlying controller. To sum up, the main contributions of this paper are as follows:

(1) In terms of control structure, the system’s 2-DOF eight-input model is converted into four 2-DOF two-output models, and based on graph theory, the four 2-DOF two-output models are treated as four multi-agent systems, respectively, realizing model dimension reduction.

(2) In terms of the control framework, this paper abandons the traditional hierarchical centralized control framework and distributed structure control framework and adopts the integrated distributed structure control framework to reduce the complexity of the model and improve the control efficiency.

(3) In terms of the selection of the control algorithm, this paper combines the distributed model predictive control (DMPC) with sliding mode variable structure control (SMC), which not only solves the problem of slow solution speed of traditional MPC, but also improves the stability and robustness of four-wheel independent drive electric vehicles.

**Figure 1:**
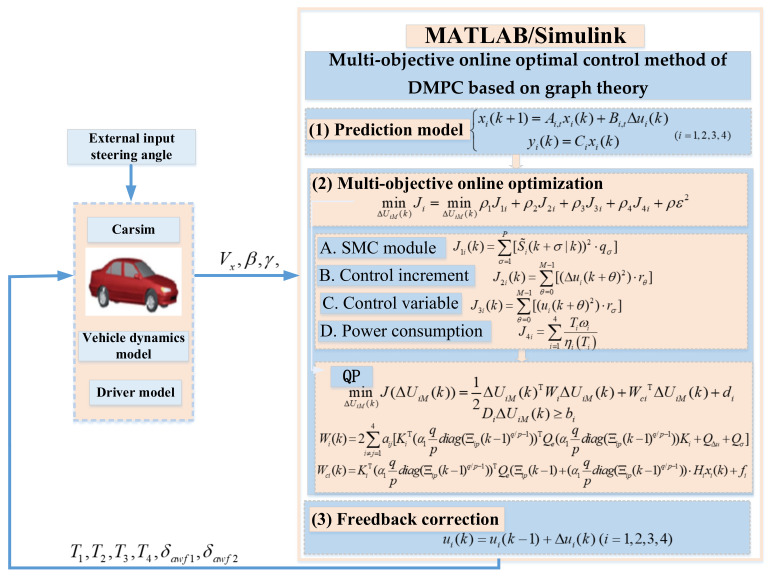
Overall structure of control system.

The overall arrangement of this article is as follows:

This paper is based on the coordinated optimization control method of AFS and DYC for four-wheel independent drive electric vehicles of MAS. Section 2 adopts an integrated distributed control framework and abandons the traditional centralized hierarchical control framework. The four-wheel independent drive electric vehicle dynamics model and the four-wheel independent drive electric vehicle dynamics reference model are established. Through vector transformation, the yaw rate at the center of mass and the sideslip angle of the center of mass are decoupled, and the vehicle 2-DOF eight-input model is transformed into four 2-DOF two-input models. In the third section, the dimensionality reduction system is regarded as four agents. According to the hardware connection structure and communication topology of the four-wheel independent drive electric vehicle, a dimensionality reduction model of 4WIDEV AFS and DYC coordinated optimal control is established based on graph theory. In the fourth section, combining SMC and DMPC, for the deviation of the vehicle yaw rate and centroid sideslip angle from their ideal values, a discrete dynamic sliding mode surface function is proposed for the agent to improve the robustness of the system against parameter changes and disturbances. The objective function considers the stability and economy of 4WIDEV and proposes an optimal control method for active front wheel steering and driving torque based on SMC and DMPC. The overall structure of the control system is shown in Figure 1. Finally, in Section 5, a hardware-in-the-loop (HIL) test bench is built for experimental verification.

Among them, $T_{1},T_{2},T_{3}$, and $T_{4}$ are the driving torque of the four wheels of the electric vehicle; $\delta_{awf1}$ and $\delta_{awf2}$ are the active left front wheel angle and the active right front wheel steering angle, respectively; $V_{x}$ is the vehicle longitudinal speed, β is the vehicle centroid sideslip angle, and γ is the vehicle yaw rate.

## 2. Dimensionality Reduction of 4WIDEV Dynamics Model Based on Vector Transformation

### 2.1. Four-Wheel Independent Drive Electric Vehicle Dynamics Model

**Figure 2:**
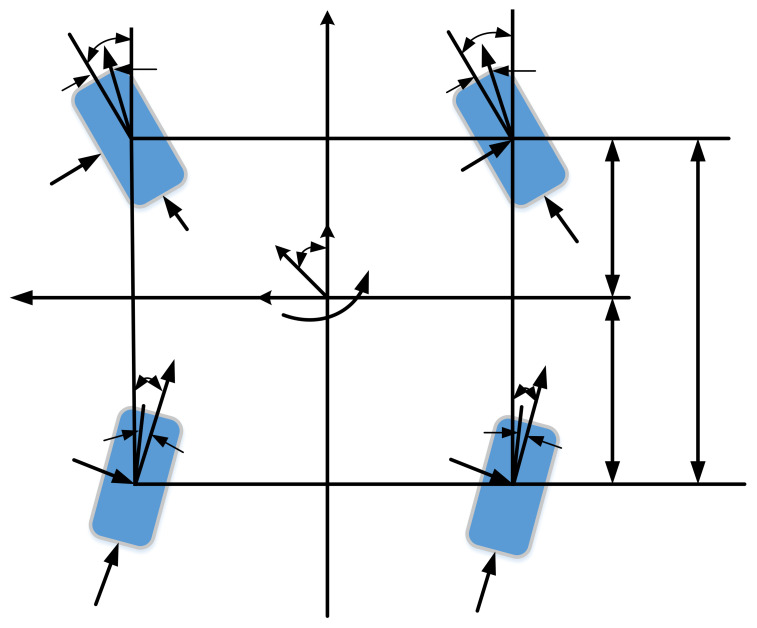
Vehicle model.

Assuming that the longitudinal and lateral speeds of the vehicle are basically unchanged, and the tire cornering characteristics are in the linear range, the roll, pitch, and vertical motions are ignored, and only the lateral, longitudinal, and yaw motions of the vehicle are considered. The vehicle dynamics model is shown in Figure 2. 

Body dynamics model based on yaw stability:

(1) $$\left\{\begin{array}{c} \dot{\beta }=\frac{1}{mv_{x}}\sum_{i=1}^{4}F_{yi}-\gamma \\ \dot{\gamma }=\frac{1}{I_{z}}[l_{1}(F_{y1}+F_{y2})-l_{2}(F_{y3}+F_{y4})+(T_{1}+T_{3}-T_{2}-T_{4})] \end{array}\right.$$

where β is the centroid slip angle, γ is the yaw rate, and $l_{1}$ and $l_{2}$ are the distances from the front/rear axles to the center of mass, respectively; $F_{yi}(i=1,2,3,4)$ represents the y-axis component of the lateral force of the ith tire, $v_{x}$ is the lateral velocity of the vehicle center of mass around the longitudinal axis, and m and $I_{z}$ are the vehicle mass and the yaw moment of inertia, respectively; $T_{1},T_{2},T_{3}$, and $T_{4}$ are the driving torque of the left front wheel, right front wheel, left rear wheel and right rear wheel, respectively.

Let $\beta_{i}$ and $\gamma_{i}$ be the side slip and the yaw rate at the center of mass of the vehicle when the lateral wheel force $F_{yi}$(i=1,2,3,4) acts alone, respectively.

Satisfying:

$$\beta =\beta_{1}+\beta_{2}+\beta_{3}+\beta_{4},\gamma =\gamma_{1}+\gamma_{2}+\gamma_{3}+\gamma_{4}.$$

Order:

$${\dot{\gamma }}_{1}=(l_{1}F_{y1}+T_{1})/I_{z},{\dot{\gamma }}_{2}=(l_{1}F_{y2}+T_{2})/I_{z},{\dot{\gamma }}_{3}=(l_{2}F_{y3}+T_{3})/I_{z},\zeta_{2}=\zeta_{4}=-1$$

$${\dot{\gamma }}_{4}=(l_{4}F_{y4}+T_{4})/I_{z},{\dot{\beta }}_{1}=(F_{y1}/mv_{x})-\gamma_{1},{\dot{\beta }}_{2}=(F_{y2}/mv_{x})-\gamma_{2},{\dot{\beta }}_{3}=(F_{y3}/mv_{x})-\gamma_{3}$$

$${\dot{\beta }}_{4}=(F_{y4}/mv_{x})-\gamma_{4},\eta_{1}=\eta_{2}=l_{1},\eta_{3}=\eta_{4}=l_{2},\zeta_{1}=\zeta_{3}=1$$

Thus, Equation (1) can be rewritten in the following form:

(2) $$\left\{\begin{array}{c} {\dot{\beta }}_{i}=\frac{1}{mv_{x}}F_{yi}-\gamma_{i}\\ {\dot{\gamma }}_{i}=\frac{1}{I_{z}}\eta_{i}F_{yi}+\frac{1}{I_{z}}\zeta_{i}T_{i} \end{array}\right.\left(i=1,2,3,4\right)$$

According to the vehicle dynamics theory, the lateral motion characteristics of the wheel are determined by the tire sideslip angle $\alpha_{i}$, which is described as:

(3) $$\begin{array}{l} \alpha_{1}\approx \delta_{1}-\arctan(\frac{v_{x}+l_{1}\gamma }{v_{x}-\frac{d}{2}\gamma })-\beta ,\alpha_{2}\approx \delta_{2}-\arctan(\frac{v_{x}+l_{1}\gamma }{v_{x}+\frac{d}{2}\gamma })-\beta \\ \alpha_{3}\approx \delta_{3}+\arctan(\frac{v_{x}-l_{2}\gamma }{v_{x}-\frac{d}{2}\gamma })-\beta ,\alpha_{4}\approx \delta_{4}+\arctan(\frac{v_{x}-l_{2}\gamma }{v_{x}+\frac{d}{2}\gamma })-\beta \end{array}$$

Since the value of 0.5dγ is very small relative to $v_{x}$, the lateral deflection angle of the four wheels is approximated as:

(4) $$\alpha_{1}\approx \delta_{1}-\frac{l_{1}\gamma }{v_{x}}-\beta ,\;\alpha_{2}\approx \delta_{2}-\frac{l_{1}\gamma }{v_{x}}-\beta ,\;\alpha_{3}\approx \delta_{3}+\frac{l_{2}\gamma }{v_{x}}-\beta ,\alpha_{4}\approx \delta_{4}+\frac{l_{2}\gamma }{v_{x}}-\beta$$

where:

$$\delta_{1}=\delta_{f}+\delta_{awf1},\delta_{2}=\delta_{f}+\delta_{awf2},\delta_{3}=\delta_{4}=0$$

where $\delta_{f}$ is the driver steering wheel angle, $\delta_{awf1}$ is the active left front wheel steering angle, and $\delta_{awf2}$ is the active right front wheel steering angle.

When the lateral acceleration of the vehicle is within 0.4g, the tire lateral deflection stiffness is within the linear range, and the lateral deflection angle is small, the lateral deflection force at each wheel can be simplified as:

(5) $$F_{y1}=k_{1}\alpha_{1},F_{y2}=k_{2}\alpha_{2},F_{y3}=k_{3}\alpha_{3},F_{y4}=k_{4}\alpha_{4}$$

In Equation (5), $k_{1}$, $k_{2}$, $k_{3}$, and $k_{4}$ are the lateral deflection stiffness of the left front wheel, right front wheel, left rear wheel, and right rear wheel, respectively.

Substituting Equations (5) and (4) into Equation (3) yields the four-wheel independent drive electric vehicle AFS and DYC coordination control model:

(6) $$\left\{\begin{array}{c} {\dot{\beta }}_{i}=K_{\beta i}\beta_{i}+(K_{\gamma i}-1)\gamma_{i}+K_{\delta i}\delta_{f}+K_{\delta i}\delta_{awfi}\\ {\dot{\gamma }}_{i}=k_{\beta i}\beta_{i}+k_{\gamma i}\gamma_{i}+\eta_{\delta i}\delta_{f}+\eta_{\delta i}\delta_{awf1}+\eta_{Ti}T_{i} \end{array}\right.$$

Among them:

$$-\frac{k_{i}}{mv_{x}}=K_{\beta i},-\frac{k_{1}l_{1}}{mv_{x}{}^{2}}=K_{\gamma 1},-\frac{k_{2}l_{1}}{mv_{x}{}^{2}}=K_{\gamma 2},\frac{k_{3}l_{2}}{mv_{x}{}^{2}}=K_{\gamma 3},\frac{k_{4}l_{2}}{mv_{x}{}^{2}}=K_{\gamma 4},\frac{k_{i}}{mv_{x}}=K_{\delta i}\;-\frac{\eta_{i}k_{i}}{I_{z}}=k_{\beta i},-\frac{l_{1}{}^{2}k_{1}}{v_{x}I_{z}}=k_{\gamma 1},-\frac{l_{1}{}^{2}k_{2}}{v_{x}I_{z}}=k_{\gamma 2},\frac{l_{2}{}^{2}k_{3}}{v_{x}I_{z}}=k_{\gamma 3},\frac{l_{2}{}^{2}k_{4}}{v_{x}I_{z}}=k_{\gamma 4},\frac{\eta_{i}k_{i}}{I_{z}}=\eta_{\delta i},\eta_{Ti}=\frac{\zeta_{i}}{I_{z}}$$

Rotational motion of the four wheels.

(7) $$J{\dot{\omega }}_{i}=T_{i}-F_{xi}R\left(i=1,2,3,4\right)$$

Vertical load per tire.

(8) $$\begin{array}{l} F_{z1}=\frac{m}{L}[\frac{1}{2}gl_{2}-\frac{1}{2}a_{x}h+\frac{a_{y}hl_{2}}{d}],F_{z2}=\frac{m}{L}[\frac{1}{2}gl_{2}-\frac{1}{2}a_{x}h-\frac{a_{y}hl_{2}}{d}]\\ F_{z3}=\frac{m}{L}[\frac{1}{2}gl_{1}+\frac{1}{2}a_{x}h+\frac{a_{y}hl_{1}}{d}],F_{z4}=\frac{m}{L}[\frac{1}{2}gl_{1}+\frac{1}{2}a_{x}h-\frac{a_{y}hl_{1}}{d}] \end{array}$$

where, in Equations (7) and (8), $F_{zi}(i=1,2,3,4)$ represents the vertical load on the left front wheel, right front wheel, left rear wheel, and right rear wheel, respectively; h is the height of the center of mass from the ground; L is the wheelbase, $a_{y}$ is the lateral acceleration, $a_{x}$ is the longitudinal acceleration, J is the moment of inertia of the wheel, $\omega_{i}$ is the angular velocity of the wheel, and R is the tire radius.

### 2.2. Reference Model for 4WIDEV Dynamics

According to Newton’s law, the linear two-degree-of-freedom vehicle dynamics equation can be described as:

(9) $$\left\{\begin{array}{c} {\dot{\beta }}^{*}=\frac{1}{mv_{x}}\sum_{i=1}^{4}F_{yi}{}^{*}-\gamma^{*}\\ {\dot{\gamma }}^{*}=\frac{1}{I_{z}}[l_{1}(F_{y1}{}^{*}+F_{y2}{}^{*})-l_{2}(F_{y3}{}^{*}+F_{y4}{}^{*})] \end{array}\right.$$

The lateral deflection angle of the four wheels is approximately:

(10) $$\alpha_{1}{}^{*}\approx \delta_{1}{}^{*}-\frac{l_{1}\gamma^{*}}{v_{x}}-\beta^{*},\alpha_{2}{}^{*}\approx \delta_{2}{}^{*}-\frac{l_{1}\gamma^{*}}{v_{x}}-\beta^{*},\alpha_{3}{}^{*}\approx \delta_{3}+\frac{l_{2}\gamma^{*}}{v_{x}}-\beta^{*},\alpha_{4}{}^{*}\approx \delta_{4}+\frac{l_{2}\gamma^{*}}{v_{x}}-\beta^{*}$$

where:

$\delta_{1}{}^{*}=\delta_{2}{}^{*}=\delta_{f}$, $\delta_{3}{}^{*}=\delta_{4}{}^{*}=0$, $\delta_{f}$ is the driver’s steering wheel turning angle.

The lateral deflection force of each wheel can be simplified as:

(11) $$F_{y1}{}^{*}=k_{1}\alpha_{1}{}^{*},F_{y2}{}^{*}=k_{2}\alpha_{2}{}^{*},F_{y3}{}^{*}=k_{3}\alpha_{3}{}^{*},F_{y4}{}^{*}=k_{4}\alpha_{4}{}^{*}$$

Similarly, the reference model of four-wheel independent drive electric vehicle dynamics can be obtained as follows:

(12) $$\left\{\begin{array}{c} {\dot{\beta }}_{i}{}^{*}=K_{\beta i}\beta_{i}{}^{*}+(K_{\gamma i}-1)\gamma_{i}{}^{*}+K_{\delta i}\delta_{f}\\ {\dot{\gamma }}_{i}{}^{*}=k_{\beta i}\beta_{i}{}^{*}+k_{\gamma i}\gamma_{i}{}^{*}+\eta_{\delta i}\delta_{f} \end{array}\right.$$

Among them:

$$-\frac{k_{i}}{mv_{x}}=K_{\beta i},-\frac{k_{1}l_{1}}{mv_{x}{}^{2}}=K_{\gamma 1},-\frac{k_{2}l_{1}}{mv_{x}{}^{2}}=K_{\gamma 2},\frac{k_{3}l_{2}}{mv_{x}{}^{2}}=K_{\gamma 3},\frac{k_{4}l_{2}}{mv_{x}{}^{2}}=K_{\gamma 4},\frac{k_{i}}{mv_{x}}=K_{\delta i},\;-\frac{\eta_{i}k_{i}}{I_{z}}=k_{\beta i},-\frac{l_{1}{}^{2}k_{1}}{v_{x}I_{z}}=k_{\gamma 1},-\frac{l_{1}{}^{2}k_{2}}{v_{x}I_{z}}=k_{\gamma 2},\frac{l_{2}{}^{2}k_{3}}{v_{x}I_{z}}=k_{\gamma 3},\frac{l_{2}{}^{2}k_{4}}{v_{x}I_{z}}=k_{\gamma 4}\;,\frac{\eta_{i}k_{i}}{I_{z}}=\eta_{\delta i}$$

## 3. AFS and DYC Coordination Control Model for 4WIDEV Based on Graph Theory

### 3.1. Four-Wheel Independent Drive Electric Vehicle AFS and DYC Coordination Control Model

Therefore, the four-wheel independent drive electric vehicle AFS and DYC deviation models are obtained from Equation (6) minus Equation (12):

(13) $$\left\{\begin{array}{c} \Delta {\dot{\beta }}_{i}=K_{\beta i}\Delta \beta_{i}+(K_{\gamma i}-1)\Delta \gamma_{i}+K_{\delta i}\delta_{awfi}\\ \Delta {\dot{\gamma }}_{i}=k_{\beta i}\Delta \beta_{i}+k_{\gamma i}\Delta \gamma_{i}+\eta_{\delta i}\delta_{awf1}+\eta_{Ti}T_{i} \end{array}\right.$$

The equation of state of Equation (13) is given by:

(14) $$\left\{\begin{array}{c} {\dot{x}}_{si}=A_{i}x_{si}+B_{i}u_{i}\\ y_{si}=c_{i}x_{si} \end{array}\right.\left(i=1,2,3,4\right)$$

where:

$$A_{i}=\left[\begin{array}{cc} K_{\beta i} & K_{\gamma i}-1\\ k_{\beta i} & k_{\gamma i} \end{array}\right],B_{i}=\left[\begin{array}{cc} K_{\delta i} & 0\\ \eta_{\delta i} & \eta_{Ti} \end{array}\right],x_{si}=\left[\begin{array}{c} \Delta \beta_{i}\\ \Delta \gamma_{i} \end{array}\right],u_{i}=\left[\begin{array}{c} \delta_{awfi}\\ T_{i} \end{array}\right],c_{i}=\left[\begin{array}{cc} 1 & 0\\ 0 & 1 \end{array}\right]$$

### 3.2. AFS and DYC Deviation Models for Four-Wheel Independent Drive Electric Vehicles Based on Graph Theory

According to the basics of graph theory, the undirected graph G=(Ω,Π,A) associates with the set of nodes $\Omega =\left\{1,\cdots ,N\right\}$, where N represents the number of nodes, and each node represents an intelligent body. $\Pi =\left\{(i_{G},j_{G}),i_{G},j_{G}\in \Omega \right\}$ stands for edge set, $i_{G}$ and $j_{G}$ can be called neighbors.$A_{T}=[a_{i_{G}j_{G}}]\in R^{N\times N}$ is the adjacency matrix, $B=diag(b_{1},b_{2},\cdots ,b_{N})$ is the diagonal matrix, and $b_{i_{G}}>0$ represents the communication topology between leaders and followers. $D=diag(d_{1}\cdots ,d_{N})$ is the entry degree matrix. Define the Laplace matrix as $L=[l_{i_{G}j_{G}}]\in R^{N\times N}$, $L=D-A_{T}$, where:

(15) $$l_{i_{G}j_{G}}=\left\{\begin{array}{cc} -a_{i_{G}j_{G}}, & j_{G}\neq i_{G}\\ \sum_{j=1}^{N}a_{i_{G}j_{G}}, & j_{G}=i_{G} \end{array}\right.$$

**Figure 3:**
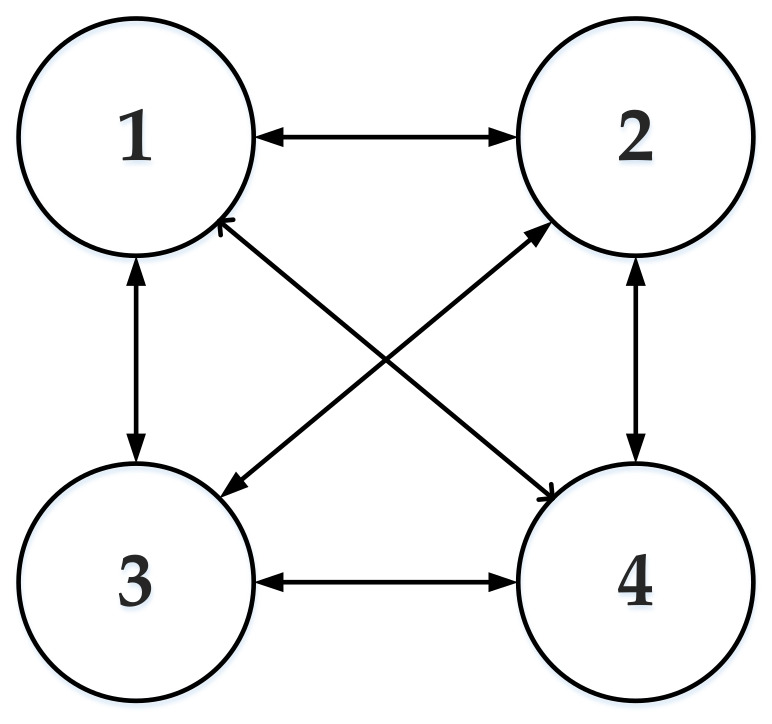
Multi-agent system topology.

In this paper, the four wheels are considered as four multi-agents. As shown in Figure 3, four agents are equivalent to four nodes in the undirected graph, and the adjacency matrix $A_{T}$, the incidence matrix D, and the Laplace matrix L of the system can be obtained based on graph theory, respectively.

$$A_{T}=\left[\begin{array}{cccc} 0 & 1 & 1 & 1\\ 1 & 0 & 1 & 1\\ 1 & 1 & 0 & 1\\ 1 & 1 & 1 & 0 \end{array}\right],D=\left[\begin{array}{cccc} 3 & 0 & 0 & 0\\ 0 & 3 & 0 & 0\\ 0 & 0 & 3 & 0\\ 0 & 0 & 0 & 3 \end{array}\right],L=D-A_{T}=\left[\begin{array}{cccc} 3 & -1 & -1 & -1\\ -1 & 3 & -1 & -1\\ -1 & -1 & 3 & -1\\ -1 & -1 & -1 & 3 \end{array}\right]$$

Therefore, Equation (14) is the equation of state for the AFS and DYC deviation models of 4WIDEV based on graph theory. Where $T_{i}$(i=1,2,3,4) represents the driving torques of the left front wheel, left rear wheel, right front wheel, and right rear wheel, respectively. $\delta_{awf1}$ is the active left front wheel steering angle, $\delta_{awf2}$ is the active right front wheel steering angle, and $\delta_{awf3}=\delta_{awf4}=0$.

## 4. Multi-Objective Online Optimal Control Method for DMPC Based on Dynamic Sliding Mode

### 4.1. AFS and DYC Coordinated Control Prediction Equation for 4WIDEV 

The Forward Euler method is used to discretize the state Equation (14) of the AFS and DYC coordinated control model of a four-wheel independent drive electric vehicle based on graph theory:

(16) $$\left\{\begin{array}{c} {\dot{x}}_{si}=\frac{x_{si}(k+1)-x_{si}(k)}{T}=A_{i}x_{s}(k)+B_{i}u_{i}(k)\\ y_{si}(k)=c_{i}x_{si}(k) \end{array}\right.$$

where T is the simulation step size.

Therefore,

(17) $$\left\{\begin{array}{c} x_{si}(k+1)=(TA_{i}+I)x_{si}(k)+TB_{i}u_{i}(k)\\ y_{si}(k)=c_{i}x_{si}(k) \end{array}\right.$$

where $I=\left[\begin{array}{cc} 1 & 0\\ 0 & 1 \end{array}\right]$.

Similarly, according to Equation (17), it is obtained that:

(18) $$\left\{\begin{array}{c} x_{si}(k)=(TA_{i}+I)x_{si}(k-1)+TB_{i}u_{i}(k-1)\\ y_{si}(k-1)=c_{i}x_{si}(k-1) \end{array}\right.$$

Let, $\Delta x_{si}=x_{si}(k+1)-x_{si}(k)$,$\Delta u_{i}(k)=u_{i}(k)-u_{i}(k-1)$; according to Equations (17) and (18), the incremental model of AFS and DYC coordinated control of four-wheel independent drive electric vehicle based on graph theory can be obtained:

(19) $$\left\{\begin{array}{c} \Delta x_{si}(k+1)=(TA_{i}+I)\Delta x_{si}(k)+TB_{i}\Delta u_{i}(k)\\ y_{si}(k+1)=c_{i}\Delta x_{si}(k+1)+y_{si}(k) \end{array}\right.$$

Let $x_{i}(k)={\left[{\begin{array}{cc} \Delta x_{si}{\left(k\right)}_{2\times 1} & y_{si}(k) \end{array}}_{2\times 1}\right]}^{T}$; the graph theory-based AFS and DYC coordinated control incremental model of four-wheel independent drive electric vehicle (19) be rearranged into the following form:

(20) $$\left\{\begin{array}{c} x_{i}(k+1)=A_{i,t}x_{i}(k)+B_{i,t}\Delta u_{i}(k)\\ y_{i}(k)=C_{i}x_{i}(k) \end{array}\right.\left(i=1,2,3,4\right)$$

where:

$$A_{i,t}={\left[\begin{array}{cc} {\left(TA_{i}+I\right)}_{2\times 2} & 0_{2\times 2}\\ c_{i}{\left(TA_{i}+I\right)}_{2\times 2} & I_{2\times 2} \end{array}\right]}_{4\times 4},B_{i,t}={\left[\begin{array}{c} TB_{i}{}_{2\times 2}\\ c_{i}TB_{i2\times 2} \end{array}\right]}_{4\times 2},C_{i}={\left[{\begin{array}{cc} 0_{2\times 2} & I \end{array}}_{2\times 2}\right]}_{2\times 4}$$

Assuming that the current moment is the k moment, the predicted time domain of the system is P and the control time domain is M, so that the predicted values of the output of the system for the next P moments under the action of M successive controls $\Delta u_{i}(k),\Delta u_{i}(k+1),\cdots ,\Delta u_{i}(k+M-1)$ are:

(21) $$\left\{\begin{array}{c} y_{i}(k+1|k)=C_{i}A_{i,t}x_{i}(k|k)+C_{i}B_{i,t}\Delta u_{i}(k)\\ y_{i}(k+2|k)=C_{i}A_{i,t}{}^{2}x_{i}(k|k)+C_{i}A_{i,t}B_{i,t}\Delta u_{i}(k)+C_{i}B_{i,t}\Delta u_{i}(k+1)\\ \vdots \\ y_{i}(k+P|k)=C_{i}A_{i,t}{}^{P}x_{i}(k|k)+C_{i}A_{i,t}{}^{P-1}B_{i,t}\Delta u_{i}(k)+\cdots +C_{i}A_{i,t}{}^{P-M}B_{i,t}\Delta u_{i}(k+M-1) \end{array}\right.$$

Order:

$$Y_{iP}(k)={\left[y_{i}(k+1|k),y_{i}(k+2|k),\ldots ,y_{i}(k+\sigma |k),\ldots ,y_{i}(k+P|k)\right]}^{T},\sigma =1,2,\ldots ,P$$

$$\Delta U_{iM}(k)=[\begin{array}{cccc} \Delta u_{i}(k) & \Delta u_{i}(k+1) & \cdots & \Delta u_{i}(k+M-1){]}^{T} \end{array}$$

According to Equation (21), the multi-step output prediction equation of the four-wheel independent drive electric vehicle AFS and DYC coordinated control model based on graph theory can be obtained:

(22) $$Y_{iP}(k)=H_{i}x_{i}(k|k)+K_{i}\Delta U_{iM}(k)$$

where:

$$H_{i}=\left[\begin{array}{c} C_{i}A_{i,t}\\ C_{i}A_{i,t}{}^{2}\\ C_{i}A_{i,t}{}^{3}\\ \vdots \\ C_{i}A_{i,t}{}^{P} \end{array}\right],K_{i}=\left[\begin{array}{ccccc} C_{i}B_{i,t} & & & & \\ C_{i}A_{i,t}B_{i,t} & C_{i}B_{i,t} & & & \\ C_{i}A_{i,t}{}^{2}B_{i,t} & C_{i}A_{i,t}B_{i,t} & C_{i}B_{i,t} & & \\ \vdots & & & & \\ C_{i}A_{i,t}{}^{P-1}B_{i,t} & C_{i}A_{i,t}{}^{P-2}B_{i,t} & C_{i}A_{i,t}{}^{P-3}B_{i,t} & \cdots & C_{i}A_{i,t}{}^{P-M}B_{i,t} \end{array}\right]$$

where $H_{i}$ and $K_{i}$ are the coefficient matrices of the multi-step output prediction in Equation (21), and the superscript “k+σ|k“ indicates the prediction of future values σ using the current values at k and σ=1,2,…,P.

### 4.2. Objective Function and Constraints of Coordinated Control of AFS and DYC for 4WIDEV Based on Dynamic Sliding Mode

Assume that the current time is k moment, and $y_{i}{}^{ref}(k+\sigma |k)$ is the ideal value of the output prediction of the system at σ moment in the future. Define the ideal value of multi-step output prediction of DYC system in the future P moment based on graph theory as:

(23) $$Y_{iP}{}^{ref}(k)={\left[y_{i}{}^{ref}(k+1|k),y_{i}{}^{ref}(k+2|k),\ldots ,y_{i}{}^{ref}(k+\sigma |k),\ldots ,y_{i}{}^{ref}(k+P|k)\right]}^{T}$$

Let $e_{i}(k+\sigma |k)=y_{i}(k+\sigma |k)-y_{i}^{ref}(k+\sigma |k)$, the multi-step output prediction error of the system at the next P moments in the future is defined as:

(24) $$E_{iP}(k)=Y_{iP}(k)-Y_{iP}{}^{ref}(k)={\left[e_{i}(k+1|k),e_{i}(k+2|k),\ldots ,e_{i}(k+\sigma |k),\ldots ,e_{i}(k+P|k)\right]}^{T}$$

Considering the influence of the state information of the adjacent jth agent on the output of its agent *i*, let:

(25) $$\varepsilon_{i}(k+\sigma |k)=\sum_{i\neq j=1}^{4}a_{ij}(e_{i}(k+\sigma |k)-e_{j}(k+\sigma |k))$$

(26) $$\Xi_{ip}(k)={\left[\varepsilon_{i}(k+1|k),\varepsilon_{i}(k+2|k),\ldots ,\varepsilon_{i}(k+\sigma |k),\ldots ,\varepsilon_{i}(k+P|k)\right]}^{T}$$

where $a_{ij}$ is an element of the adjacency matrix $A_{T}$. 

According to Equations (24)–(26):

(27) $$\Xi_{ip}(k)=\sum_{i\neq j=1}^{4}a_{ij}(E_{iP}(k)-E_{jP}(k))$$

Define the following discrete slip surfaces:

(28) $${\tilde{S}}_{i}(k+\sigma |k)=\varepsilon_{i}(k+\sigma -1|k)+\alpha_{1}\frac{q}{p}\varepsilon_{i}{\left(k+\sigma -1|k\right)}^{q/p-1}\varepsilon_{i}(k+\sigma |k)\;\sigma =1,2,\ldots ,P$$

where p and q are positive odd numbers, and $1<q/p<2,\alpha_{1}>0$.

Let, ${\tilde{S}}_{iP}(k)={\left[{\tilde{S}}_{i}(k+1|k),{\tilde{S}}_{i}(k+2|k),\ldots ,{\tilde{S}}_{i}(k+\sigma |k),\ldots {\tilde{S}}_{i}(k+P|k)\right]}^{T}$, according to Equations (27) and (28):


(29) $$\begin{array}{c} {\tilde{S}}_{ip}\left(k\right)=\Xi_{ip}\left(k-1\right)+\alpha_{1}\frac{q}{p}\mathrm{diag}\left(\Xi_{ip}{\left(k-1\right)}^{q/p-1}\right)\Xi_{ip}\left(k\right)\\ =\Xi_{ip}\left(k-1\right)+\alpha_{1}\frac{q}{p}\mathrm{diag}\left(\Xi_{ip}{\left(k-1\right)}^{q/p-1}\right)\sum_{i\neq j=1}^{4}a_{ij}\left(E_{iP}\left(k\right)-E_{jP}\left(k\right)\right)\\ =\Xi_{ip}\left(k-1\right)+\alpha_{1}\frac{q}{p}\mathrm{diag}\left(\Xi_{ip}{\left(k-1\right)}^{q/p-1}\right)\sum_{i\neq j=1}^{4}a_{ij}\left(Y_{iP}\left(k\right)-Y_{iP}^{ref}\left(k\right)-Y_{jP}\left(k\right)+Y_{jP}^{ref}\left(k\right)\right)\\ =\Xi_{ip}\left(k-1\right)+\alpha_{1}\frac{q}{p}\mathrm{diag}\left(\Xi_{ip}{\left(k-1\right)}^{q/p-1}\right)\sum_{i\neq j=1}^{4}a_{ij}\left[H_{i}x_{i}\left(k|k\right)+K_{i}\Delta U_{iM}\left(k\right)-H_{j}x_{j}\left(k|k\right)-K_{j}\Delta U_{jM}\left(k\right)\right] \end{array}$$

(1) The four agents can follow the ideal values of the vehicle yaw rate and the center of mass sideslip angle, and the mutual influence of the four agents is minimized. Taking into account the vehicle stability and system robustness, the performance index $J_{1i}$ of the discrete dynamic sliding mode surface function is defined:

(30) $$J_{1i}(k)=\sum_{\sigma =1}^{P}{\left[{\tilde{S}}_{i}(k+\sigma |k)\right)}^{2}\cdot q_{\sigma }]$$

where k is the current moment, P is the predicted time domain, and $q_{\sigma }$ is the weighting factor; let $Q_{e}=diag(q_{1},q_{2},\cdots ,q_{P})$, which is the weighting matrix of the output variables, and $a_{ij}$ is the element of the adjacency matrix $A_{T}$.

(2) To improve the economy of the vehicle and reduce the energy loss of the four-wheel independent drive electric vehicle, we hope that the energy loss of the actuator is as small as possible. The multi-objective optimization objective function based on dynamic sliding mode is defined as:

(a) In the whole process, the control action is as small as possible to reduce energy loss and take into account the economy of the entire vehicle system. Define performance index $J_{2i}$:

(31) $$J_{2i}(k)=\sum_{\theta =0}^{M-1}\left[(\Delta u_{i}{\left(k+\theta \right)}^{2})\cdot r_{\theta }\right]$$

where k is the current moment, M is the control time domain, θ=0,1,2,⋯,M−1, $\Delta u_{i}$ is the amount of change in the control quantity, $r_{\theta }$ is the weighting factor, and let $Q_{\Delta u}=diag(r_{0},r_{1},\cdots ,r_{M-1})$ be the weighting matrix of the control increment.

(b) Minimize tire utilization (maximize tire stability margin). 

Tire utilization is the ratio of the actual road adhesion to the maximum road adhesion it can obtain, and it characterizes the stability margin of the tire.


(32) $$\begin{array}{c} \lambda_{r}=\sum_{i=1}^{4}\frac{F_{xi}^{2}+F_{yi}^{2}}{{\left(\mu F_{zi}\right)}^{2}}\left(i=1,2,3,4\right) \end{array}$$

Since the tire lateral forces are uncontrollable, the control allocation in this paper considers only the tire longitudinal forces. Therefore, the optimization objective function $J_{3i}$ is used to minimize the control energy consumption and to ensure the maximum tire stability margin (minimum tire utilization).

(33) $$J_{3i}(k)=\sum_{\theta =0}^{M-1}\left[(u_{i}{\left(k+\theta \right)}^{2})\cdot r_{\sigma }\right]$$

where $r_{\sigma }=\frac{1}{\mu RF_{zi}}$ is the weighting factor, and let $Q_{\sigma }=diag(r_{\sigma },r_{\sigma },\cdots ,r_{\sigma })$ be the weighting matrix of the control increment.

(c) Minimization of power consumption of the drive system

The total power expression of the in-wheel motor of the four-wheel independent drive electric vehicle is as follows [26]:

(34) $$P=\sum_{i=1}^{4}\frac{P_{i}}{\eta_{i}\left(T_{i}\right)}=\sum_{i=1}^{4}\frac{T_{i}\omega_{i}}{\eta_{i}\left(T_{i}\right)}$$

$P_{i}$ and $\eta_{i}\left(T_{i}\right)$ represent the power and the corresponding efficiency of the motor i in the drive energy mode, respectively. From the motor map diagram, it is clear that the motor efficiency will be obtained by real-time motor torque and speed, so the fourth objective function plays an essential role in promoting motor operation in the high-efficiency region. Therefore, use the optimization objective function $J_{4i}$ to minimize the power consumption of the drive system.

(35) $$J_{4i}=f_{i}{}^{Τ}\Delta U_{iM}$$

Therefore, the objective function of the multi-objective optimal allocation algorithm based on distributed model predictive control consists of four parts: The first term satisfies the robustness of the system and ensures the handling stability of the vehicle during steering. The second term describes the constraints on the changes of the control variables, aiming at reducing the energy loss of the system and improving the system economy. The third term is designed to ensure the vehicle’s stability during steering. The fourth term focuses on achieving energy goals. The overall control objective is to save costs while keeping the vehicle safe, which is as follows:

(36) $$\underset{\Delta U_{iM}(k)}{\min}J_{i}=\underset{\Delta U_{iM}(k)}{\min}J_{1i}+J_{2i}+J_{3i}+J_{4i}+\rho \varepsilon^{2}$$

where ρ is the weighting factor and ε is the relaxation factor.

Considering the system limitations and safety, the maximum drive torque and its rate of change of the whole vehicle system are bounded as follows:

(1) Control volume constraint:

(37) $$u_{i\min}(k+\theta )\leq u_{i}(k+\theta )\leq u_{i\max}(k+\theta )\theta =0,1,2,\cdots M-1$$

where $u_{i\min}(k+\theta )$ is the minimum value of the control variable and $u_{i\max}(k+\theta )$ is the maximum value of the control variable.

It can be seen that, for θ=0,1,2,⋯M−1, formula $u_{i}(k+\theta )=\sum_{f=0}^{\theta }\Delta u_{i}(k+f)+u_{i}(k-1)$ is established. Substituting it into constraint Equation (37), we can get:

(38) $$-\sum_{f=0}^{\theta }\Delta u_{i}(k+f)\geq u_{i}(k-1)-u_{\max}(k+1)$$

(39) $$\sum_{f=0}^{\theta }\Delta u_{i}(k+f)\geq u_{i\min}(k+1)-u_{i}(k-1)$$

That is, the following matrix form is obtained:

(40) $$\left[\begin{array}{c} -L_{U}\\ L_{U} \end{array}\right]\Delta U_{iM}(k)\geq \left[\begin{array}{c} u(k-1)-u_{\max}(k)\\ \vdots \\ u(k-1)-u_{\max}(k+M-1)\\ u_{\min}(k)-u(k-1)\\ \vdots \\ u_{\min}(k+M-1)-u(k-1) \end{array}\right]$$

(2) Control of incremental constraints.

(41) $$-\Delta u_{i\min}(k+\theta )\leq \Delta u_{i}(k+\theta )\leq \Delta u_{i\max}(k+\theta )\theta =0,1,2,\cdots M-1$$

According to the above analysis, the form of control increment matrix can be obtained in the same way:

(42) $$\left[\begin{array}{c} -I_{U}\\ I_{U} \end{array}\right]\Delta U_{iM}(k)\geq \left[\begin{array}{c} -\Delta u_{\max}(k)\\ \vdots \\ -\Delta u_{\max}(k+M-1)\\ -\Delta u_{\min}(k)\\ \vdots \\ -\Delta u_{\min}(k+M-1) \end{array}\right]$$

Among them:

$$I_{U}=\left[\begin{array}{cccc} 1 & 0 & \cdots & 0\\ 0 & 1 & \cdots & 0\\ \vdots & \vdots & \cdots & \vdots \\ 0 & 0 & \cdots & 1 \end{array}\right],L_{U}=\left[\begin{array}{cccc} 1 & 0 & \cdots & 0\\ 1 & 1 & \cdots & 0\\ \vdots & \vdots & \cdots & \vdots \\ 1 & 1 & \cdots & 1 \end{array}\right]$$

Organizing Equations (36)–(42), the constraint problem can be described as:

(43) $$D_{i}\Delta U_{iM}(k)\geq b_{i}$$

Among them,

$$D_{i}=\left[\begin{array}{c} \begin{array}{c} -L_{U}\\ L_{U} \end{array}\\ \begin{array}{c} -I_{U}\\ I_{U} \end{array} \end{array}\right],b_{i}=\left[\begin{array}{c} \begin{array}{c} u(k-1)-u_{\max}(k)\\ \vdots \\ u(k-1)-u_{\max}(k+M-1)\\ u_{\min}(k)-u(k-1)\\ \vdots \\ u_{\min}(k+M-1)-u(k-1) \end{array}\\ \begin{array}{c} -\Delta u_{\max}(k)\\ \vdots \\ -\Delta u_{\max}(k+M-1)\\ -\Delta u_{\min}(k)\\ \vdots \\ -\Delta u_{\min}(k+M-1) \end{array} \end{array}\right]$$

$$u_{i\max}=\min(T_{m},\sqrt{{\left(\mu F_{zi}\right)}^{2}-F_{yi}{}^{2}R}),u_{i\min}=\max(-T_{m},-\sqrt{{\left(\mu F_{zi}\right)}^{2}-F_{yi}{}^{2}R})$$

Therefore, the objective function constraint of the prediction equation of the MAS-based four-wheel independent drive electric vehicle AFS and DYC coordinated control model is selected as the form of Equation (43).

### 4.3. Optimal Solution

Considering the constraints of the system in Equations (43), the optimization objective in Equation (36) of the system can be solved by transforming it into a standard linear quadratic programming (QP) problem with constraints.

(44) $$\underset{\Delta U_{iM}(k)}{\min}J(\Delta U_{iM}(k))=\frac{1}{2}\Delta U_{iM}{\left(k\right)}^{Τ}W_{i}\Delta U_{iM}(k)+W_{ci}{}^{Τ}\Delta U_{iM}(k)+d_{i}$$

$$s.t\;D_{i}\Delta U_{iM}(k)\geq b_{i}$$

where $\Delta U_{iM}(k)$ is the decision variable; the matrix $W_{i}$ is a Hessian matrix and symmetric positive definite matrix, which represents the quadratic part of the objective function; the vector $W_{ci}$ describes the linear part; and $d_{i}$ is independent of $\Delta U_{iM}(k)$ and is independent of the determined $\Delta U_{iM}(k)$. When the $W_{i}$ matrix is positive-definite or semi-positive-definite and the constraint is linear, the above optimization solution problem is a convex optimization problem with a unique solution. Therefore, the QP solution process is as follows:

$$W_{i}(k)=2\sum_{i\neq j=1}^{4}a_{ij}[K_{i}{}^{Τ}{\left(\alpha_{1}\frac{q}{p}diag(\Xi_{ip}{\left(k-1\right)}^{q/p-1})\right)}^{Τ}Q_{e}(\alpha_{1}\frac{q}{p}diag(\Xi_{ip}{\left(k-1\right)}^{q/p-1}))K_{i}+Q_{\Delta u}+Q_{\sigma }]$$

$$W_{ci}(k)=K_{i}{}^{Τ}{\left(\alpha_{1}\frac{q}{p}diag(\Xi_{ip}{\left(k-1\right)}^{q/p-1})\right)}^{Τ}Q_{e}(\Xi_{ip}(k-1)+(\alpha_{1}\frac{q}{p}diag(\Xi_{ip}{\left(k-1\right)}^{q/p-1}))\cdot H_{i}x_{i}(k)+f_{i}$$

From $\frac{\partial J_{i}}{\partial \Delta U_{iM}}=W_{i}\Delta U_{iM}-W_{ci}=0$, we get:

(45) $$\Delta U_{iM}(k)=W_{i}{}^{T}W_{ci}$$

After solving the model predictive control in each control cycle, the control input increment in the control time domain is obtained:

(46) $$\Delta U_{iM}{}^{*}(k)=[\begin{array}{cccc} \Delta u_{i}{\left(k\right)}^{*} & \Delta u_{i}{\left(k+1\right)}^{*} & \cdots & \Delta u_{i}{\left(k+M-1\right)}^{*}{]}^{T} \end{array}$$

The first element in the control sequence acts on the system as a control input increment, namely:

(47) $$u_{i}(k)=u_{i}(k-1)+\Delta u_{i}{\left(k\right)}^{*}$$

The system processes this control quantity predicts the output of the next cycle according to the state quantity, and obtains a new control increment sequence through optimization to scroll optimization until the system completes the control process.

## 5. Simulation Test Verification

### 5.1. CarSim Vehicle Model Building

**Figure 4:**
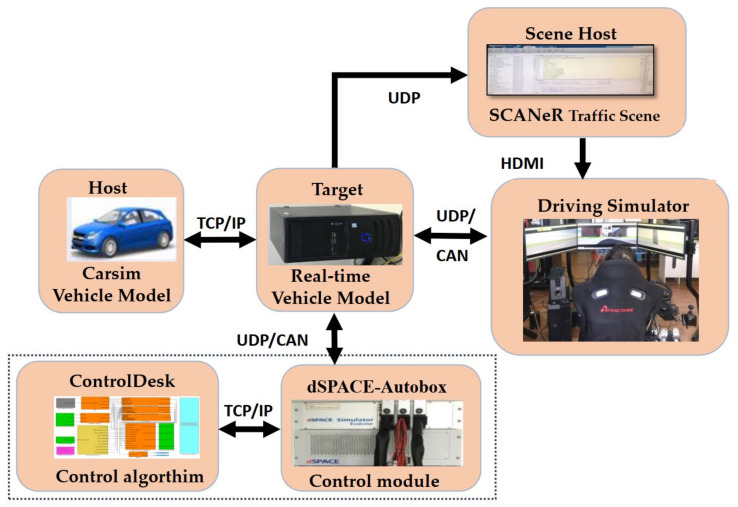
Structure of experimental platform.

**Figure 5:**
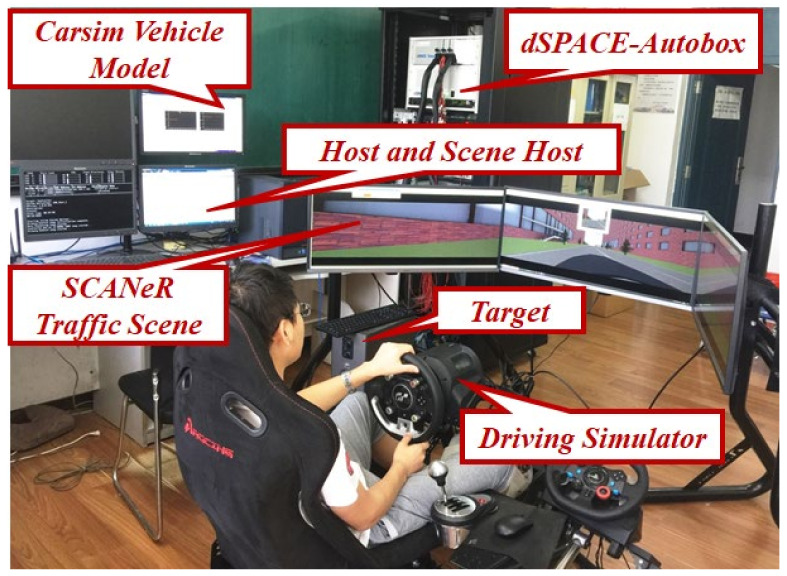
Experimental platform.

The experimental platform of this paper includes a driving simulator, dSPACE for running the control algorithm, a host for running the Carsim vehicle model and SCANR traffic scene model, and a target machine for running the real-time vehicle model. The driver operates the steering wheel and pedal simulator to generate the steering wheel angle and driving pedal signals while driving. After the CAN turns the Ethernet module to the target, use the demand of the real-time vehicle model to calculate the yaw rate, centroid sideslip angle, and longitudinal speed of the four wheels and send them to the DMPC Autobox control module, obtain the four-wheel torque control signals and send them to the target machine, and calculate to obtain the vehicle position change. The driver controls the vehicle according to the change of the scene from the Ethernet to the traffic scene and the vehicle attitude change scene. Then, form a closed loop structure. The structure of the experimental platform is shown in Figure 4, and the experimental platform is shown in Figure 5.

Next, build a vehicle model in CarSim, use pacejka 5.2 [27] for the tire model, change the vehicle drive mode to direct drive by in-wheel motor, and change the torque to be input from the external model to the wheel. Build a drive torque controller in Simulink directly to provide power for the vehicle, set the Carsim input variables as four-wheel drive torques, and select the vehicle model in the CarSim vehicle model library as B-Class, sports; the vehicle model data are shown in Table 1.

### 5.2. Constant Speed Turning Conditions

**Figure 6:**
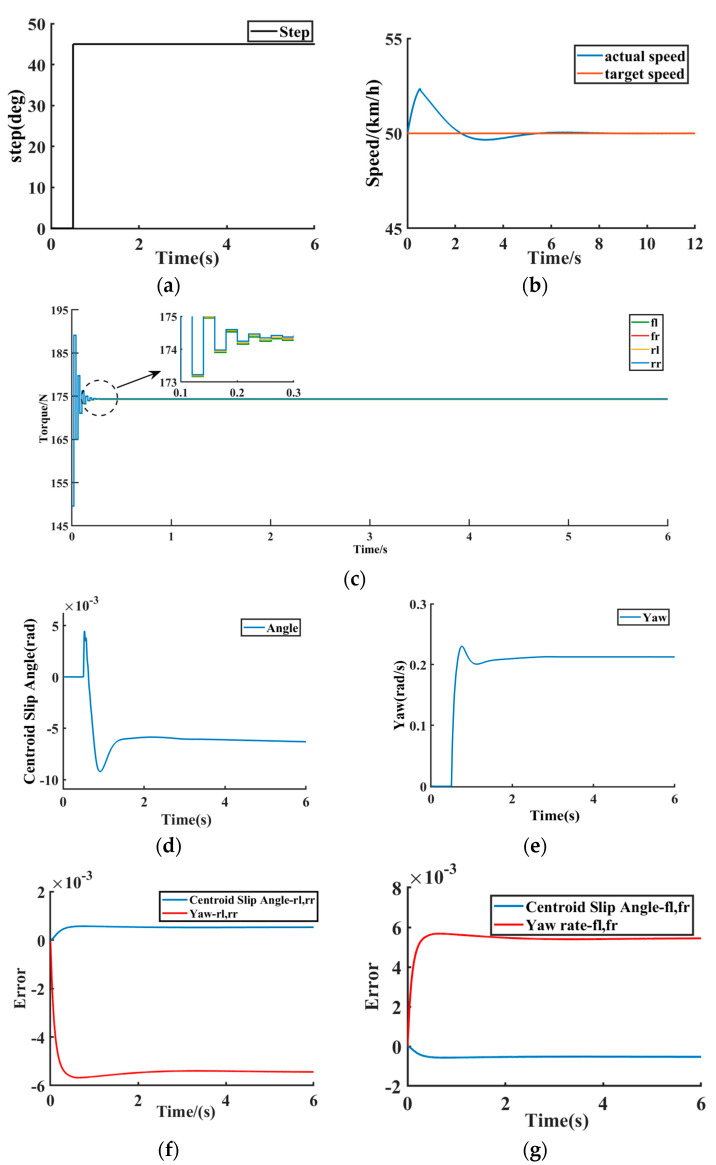

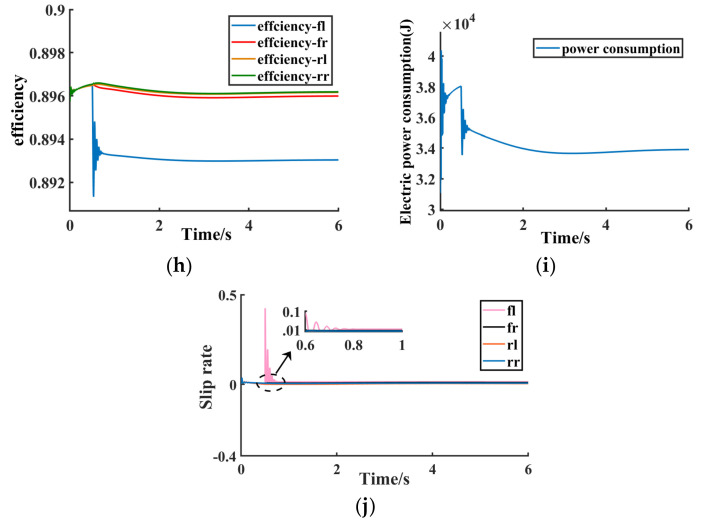
Constant speed turning condition. (**a**) Vehicle front wheel turning angle. (**b**) Vehicle speed and target vehicle speed. (**c**) Driving torque of the wheels. (**d**) Vehicle center of mass side slips angle. (**e**) Vehicle yaw rate. (**f**) rl, rr error. (**g**) fl, fr error. (**h**) Motor efficiency. (**i**) Electric power consumption. (**j**) Wheel slip rate.

When turning at a constant speed, set the road adhesion coefficient to 0.8, set the initial speed to 50 km/h, and input a steering wheel turning angle of 45-degree steps to the vehicle at 0.5 s. The simulation results under this condition are shown in Figure 6a–j.

Figure 6a−j show the simulation results of the DMPC multi-objective online optimization control method based on the dynamic sliding mode proposed in this paper under the condition of constant speed turning. Figure 6a shows the size of the steering angle under the condition of constant speed turning. Figure 6b shows the vehicle speed and the vehicle target speed. Figure 6c shows the driving torque based on the method proposed in this paper. It can be seen that the driving torque quickly converges to about 174 Nm after 0.01 s of oscillation. Although there is some overshoot within 0.01 s, based on the proposed method compared with the traditional MPC and layered control methods, the DMPC method has a strong rapidity, which improves the dynamic performance and computational efficiency of the system. Figure 6d reflects that the vehicle center of mass slip angle converges to −0.006 rad around 1.5 s, and Figure 6e reflects that the vehicle yaw rate converges to 0.2 rad/s around 1.5 s. Figure 6d,e prove that the proposed method guarantees vehicle stability. Figure 6f,g are the yaw rate deviation value and the sideslip angle deviation value of the right front wheel, right rear wheel, left front wheel, and left rear wheel agent, respectively. Figure 6h shows the efficiency of the hub motor of the four-wheel independent drive electric vehicle. It can be seen that the efficiency of the four wheels remains stable after the fluctuation of 0.5 s, which proves the effectiveness of the control method in this paper. Figure 6i shows the power consumption of the four-wheel independent drive electric vehicle. It can be seen that the power consumption of the motor is gradually reduced, which proves the effectiveness of the control method in this paper. Figure 6j shows the slip rate of the wheel, where the slip rate fluctuates slightly at about 0.5−0.7 s and the wheel slip rate converges to around 0.01 after 0.7 s, indicating that the control method proposed in this paper can ensure that the stable driving of the vehicle does not slip. Therefore, according to the simulation results shown in Figure 6a−j, the 4WIDEV AFS and DYC coordinated optimal control method based on the MAS model proposed in this paper effectively balances the system dynamic performance, computational efficiency, and vehicle economy.

### 5.3. Accelerated Turning Conditions

**Figure 7:**
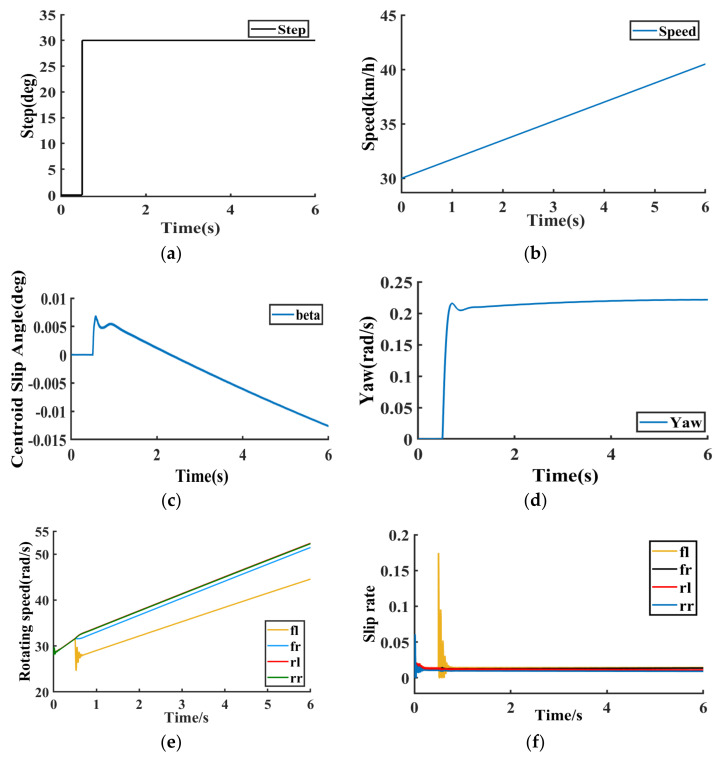

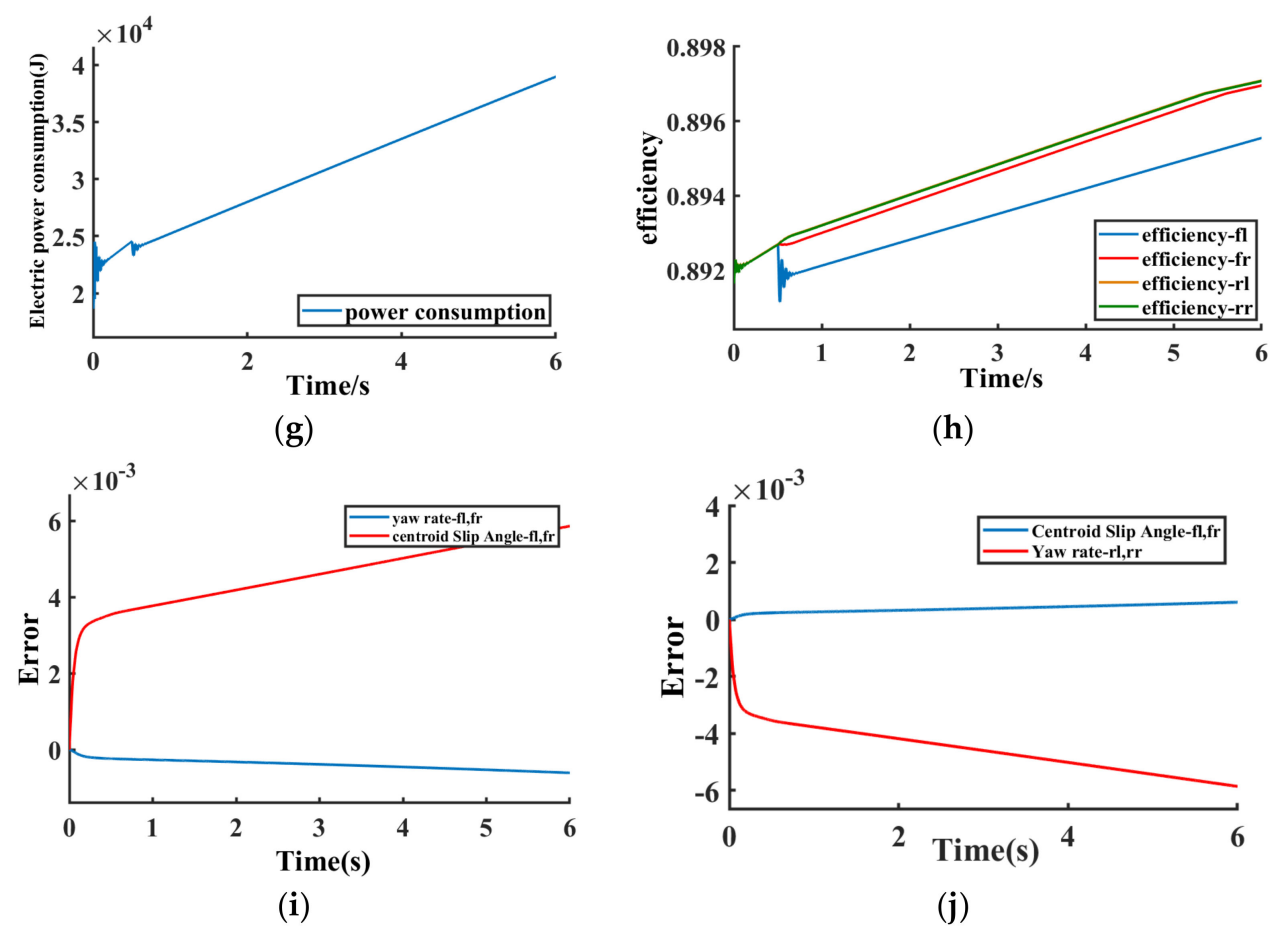
Accelerating turning condition. (**a**) Vehicle front wheel angle. (**b**) Vehicle speed. (**c**) Vehicle center of mass side slip angle. (**d**) Vehicle yaw rate. (**e**) Wheel speed. (**f**) Wheel slip rate. (**g**) Electric power consumption. (**h**) Efficiency. (**i**) fl, fr error. (**j**) rl, rr error.

When accelerating the vehicle during the turn, the road adhesion coefficient is 0.8, the initial vehicle speed is 30 km/h, the vehicle accelerates at 2 m/s^2^, and 30 degrees is the input to the steering wheel corner at 0.5 s. The simulation results under this operating condition are shown in Figure 7a−j.

Figure 7a−j show the simulation results of the DMPC multi-objective online optimization control method based on the dynamic sliding mode proposed in this paper under accelerated turning conditions. Figure 7a is the steering angle of the left front wheel of the vehicle under accelerated turning conditions. Figure 7b is the actual speed of the vehicle. Figure 7c reflects the vehicle’s center of mass slip angle within the vehicle stability range. Figure 7d reflects that the vehicle yaw rate converges to 0.2 rad/s in about 0.8 s. Figure 7c,d prove that the control method proposed in this paper can ensure vehicle stability under vehicle acceleration and steering conditions. Figure 7e is the speed of the four wheels. Figure 7f is the slip rate of the four wheels, and the slip rate fluctuates slightly at about 0.5−0.6 s, and the wheel slip rate converges to around 0.01 after 0.6 s, which proves that the control method proposed in this paper ensures that the vehicle runs stably without slipping. Figure 7g shows the electric power consumption of the four in-wheel motors under accelerated turning conditions. Figure 7h shows the motor efficiency of four-wheel independent drive electric vehicles. Under the control method in this paper, the motor efficiency gradually rises, which proves the effectiveness of the control method in this paper. Figure 7i,j are the yaw rate deviation value and the center of mass slip angle deviation value of the vehicle’s right front wheel, right rear wheel, left front wheel, and left rear wheel, respectively. To sum up, according to the simulation results in Figure 7a−j, it is shown that the MAS model-based 4WIDEV AFS and DYC coordinated optimal control method proposed in this paper effectively balances the system dynamic performance, computational efficiency, and the economy of the vehicle.

### 5.4. DLC Maneuver on Slippery Road

**Figure 8:**
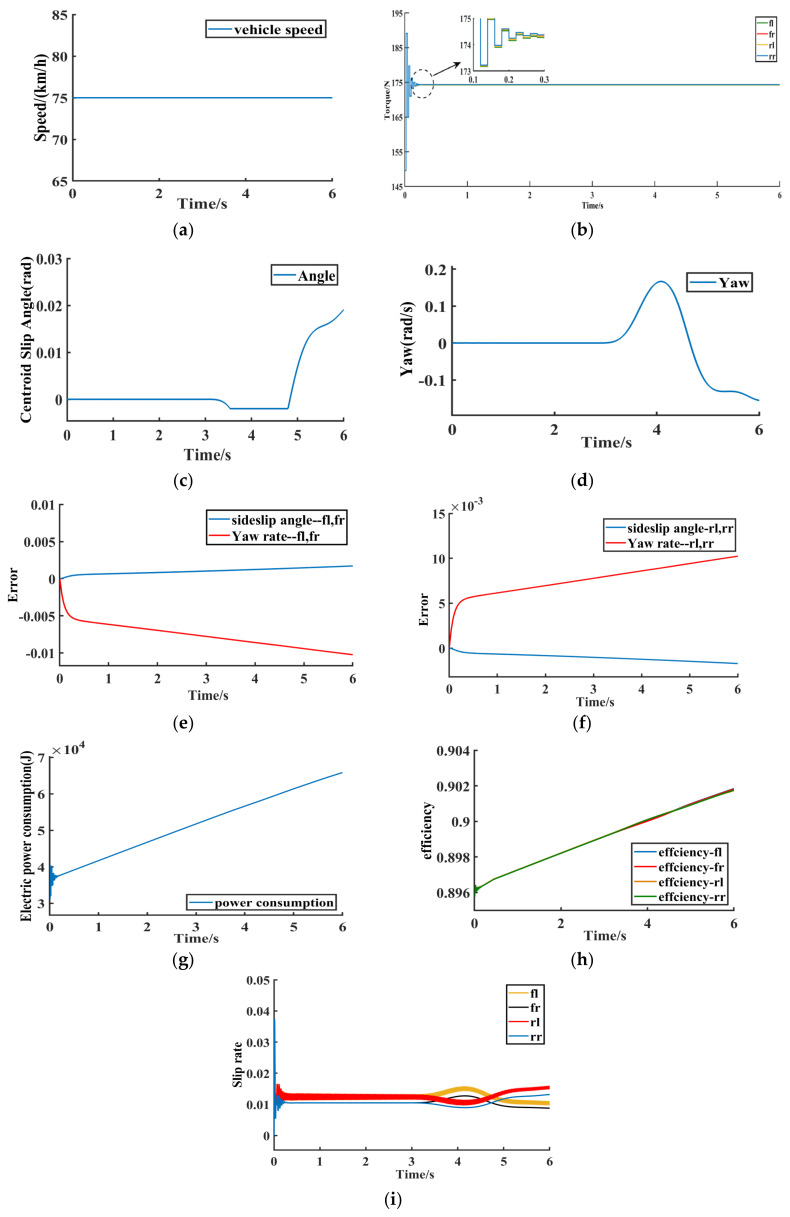
DLC. (**a**) Vehicle target speed. (**b**) In-wheel motor torque. (**c**) Vehicle sideslip angle. (**d**) Yaw rate. (**e**) fl, fr error. (**f**) rl, rr error. (**g**) Motor efficiency. (**h**) Electric power consumption. (**i**) Wheel slip rate.

The low adhesion road with an adhesion coefficient of 0.3 is selected, and the simulation experiment of double lane shifting is carried out at the target speed of 72 km/h. The simulation results are shown in Figure 8a−i.

The simulation results under this working condition are shown in Figure 8a–i. Figure 8a−i show the simulation results of the dynamic sliding model-based DMPC multi-objective online optimal control method proposed in this paper under the double-shifted line condition. Figure 8a shows the target vehicle speed of the four-wheel independent drive electric vehicle. Figure 8b shows the drive torque of the four-wheel independent drive electric vehicle under the proposed control method. It can be seen that the drive torque oscillates slightly at t=0.3s and then converges quickly to 174 Nm with a peak time of 0.1 s. With the control method of DMPC proposed in this paper, the drive torque convergence is extremely fast, and compared with the traditional centralized control method in the literature [26], the control method in this paper makes the drive torque convergence speed increase 32.33 times compared with the literature [26].

In Figure 8e, the red curve is the deviation value between the actual yaw rate and the ideal yaw rate of the left front wheel and the left rear wheel of the vehicle, and the blue curve is the deviation value between the actual centroid sideslip angle and the ideal centroid sideslip angle of the left front wheel and the left rear wheel of the vehicle. In Figure 8f, the red curve is the deviation value between the actual yaw rate and the ideal yaw rate of the right front wheel and the right rear wheel of the vehicle, and the blue curve is the deviation value between the actual centroid sideslip angle and the ideal centroid sideslip angle of the right front wheel and the right rear wheel of the vehicle.

Figure 8g shows the wheel motor electric consumption under the double-shifted line condition, and using the same economic index conditions as in the literature [26], the wheel motor electric consumption in this paper is 65.8 J. Compared with the literature [26], the wheel motor electric consumption is reduced by 16.6%, and the proposed method in this paper reduces the model complexity, improves the computational efficiency, and weighs the system dynamic performance based on ensuring vehicle stability. Figure 8h shows the motor efficiency, so with the control method in this paper, the electric consumption of the four hub motors is reduced and the motor efficiency keeps rising. Figure 8i shows the wheel slip rate, and the wheel slip rate is stable between 0.01 and 0.02, which proves that the proposed control method in this paper can ensure the stable driving of the vehicle without skidding. Therefore, according to the simulation results of Figure 8a−i, the convergence speed of the drive torque is improved by 32.33 times, and the electric consumption of the wheel motor is reduced by 16.6% under the coordinated optimal control method of 4WIDEV AFS and DYC based on the MAS model proposed in this paper compared with the literature [26]. The method effectively weighs the dynamic performance of the system and improves the computational efficiency and the overall vehicle economy.

## 6. Conclusions

In this paper, we adopt an integrated distributed control structure, abandon the traditional centralized hierarchical control framework, and propose a MAS-based coordinated and optimized control method for four-wheel independent drive electric vehicles AFS and DYC, which realizes model dimensionality reduction and is suitable for engineering applications. In the control method part of this paper, SMC is combined with DMPC, and the dynamic sliding mode surface function is introduced in the objective function to improve the robustness of the system when coping with parameter changes and disturbances, weighing the dynamic performance of the system, and improving the computational efficiency. Compared with the traditional centralized control method, the torque solution speed of the control method proposed in this paper is increased by 32.33 times, and the power consumption of the hub motor is reduced by 16.6%. However, the research content of this paper only considers the state of the four-wheel independent drive electric vehicle understeering, starting, and double-lane change conditions, and the steering angle is within the range of 0–5°. In the future, the driving state of the vehicle under more conditions can be considered.

## Figures and Tables

**Table 1:** Basic parameters of the whole vehicle.

Parameters	Value
Vehicle mass m/kg	1500
Wheelbase L/m	2.33
Track width d/m	1.48
Distance from centroid to front axle $l_{1}/m$	1.165
Distance from centroid to rear axle $l_{2}/m$	1.155
Centroid height $h_{g}/m$	0.375
Wheel radius r/m	0.33
Peak torque N/m	600

## Data Availability

Data are contained within the article.
